# More Accurate Insight into the Incidence of Human Rabies in Developing Countries through Validated Laboratory Techniques

**DOI:** 10.1371/journal.pntd.0000765

**Published:** 2010-11-30

**Authors:** Laurent Dacheux, Supaporn Wacharapluesadee, Thiravat Hemachudha, François-Xavier Meslin, Philippe Buchy, Jean-Marc Reynes, Hervé Bourhy

**Affiliations:** 1 Institut Pasteur, Dynamics and Host Adaptation Unit, National Reference Centre for Rabies, WHO Collaborative Centre for Reference and Research on Rabies, Paris, France; 2 Molecular Biology Laboratory for Neurological Diseases, Department of Medicine, Chulalongkorn University Hospital, Bangkok, Thailand; 3 Neglected Zoonotic Diseases, Department of Neglected Tropical Diseases, Cluster HIV/AIDS, Malaria, Tuberculosis and Neglected Tropical Diseases (HTM), WHO Headquarters, Geneva, Switzerland; 4 Institut Pasteur du Cambodge, Virology Unit, Cambodia; 5 Centre Pasteur du Cameroun, Virology Unit, Cameroon; New York Blood Center, United States of America

## How to Improve Human Rabies Diagnosis in Developing Countries

Over the past 10 years, new techniques and protocols have been proposed for rabies diagnosis, especially in humans. However, the reported number of laboratory-confirmed human rabies cases remains limited and underestimates the real impact of this neglected zoonotic disease, particularly in enzo-epizootic areas of Asia and Africa [1]. The regular reporting of diagnostic data through the structures of the World Organization for Animal Health and the World Health Organization (WHO) (using the RABNET network, for example), together with the importance of the status of rabies as a notifiable disease in all countries, remain crucial for the surveillance and control of rabies, especially in humans. There is clearly a need to improve diagnostic tools appropriate for the facilities in these countries. This paper provides a review of recent publications relating to the evaluation of methods for human rabies diagnosis in developing country settings in order to highlight the sampling methods and techniques that are likely to give the most reliable results.

In their recent review entitled “Emerging technologies for the detection of rabies virus: challenges and hopes in the 21st century”, Fooks et al. [2] provide information on some of the latest techniques to detect rabies virus or nucleic acid in diagnostic samples. However, additional major techniques not mentioned by Fooks et al. [2] are also available, some concerning recent studies addressing the broader challenge of human rabies diagnosis in developing countries, which we aim to describe here.

## A Key Step: Selection of Biological Samples

Several biological samples can be used for intra-vitam diagnosis of human rabies: saliva, cerebral spinal fluid (CSF), [2] but also skin biopsies containing hair follicles collected at the nape of the neck, extracted hair follicles, tears, and urine [3]–[5]. The collection of brain biopsy samples [6] should be discouraged for intra-vitam diagnosis and should be restricted to post-mortem diagnosis. Skin biopsies can also be used for the post-mortem diagnosis of rabies encephalitis to replace brain biopsy when the latter is not feasible (which is the case in most of enzootic areas in Asia and Africa where cerebral brain biopsies are rarely authorized by the patient's family) [3], [7]. Because the viral shedding in saliva, CSF, and urine is suggested to be intermittent, multiple different samples (e.g., skin, saliva, urine) must be collected and analyzed by molecular methods for an intra-vitam rabies diagnosis. However, the sensitivity of testing only one skin biopsy per patient has been shown to be ≥98% [3], and it is possible to achieve maximal sensitivity (up to 100%) for furious rabies encephalitis by collecting and testing three serial daily saliva samples per patient [3]. Viral RNA detection from paralytic rabies patients is also possible, although the sensitivity may be lower. In a recent study conducted in Thailand, the sensitivity of detection of paralytic and furious rabies was 50% (*n* = 6) and 92% (*n* = 50) per patient, respectively [4], [5]. A summary of the sensitivity results obtained for different biological samples using molecular methods is shown in Table 1. Two sets of recommendations for sample collection in current use in reference laboratories in both developed and developing countries are shown in Tables 2 and 3.

**Table 1:** Description of Biological Samples Analyzed for Molecular Diagnosis of Human Rabies in Large Cohorts of Suspected or Confirmed Rabid Patients in Local Laboratory Conditions.

Samples	Technique (Target Gene)	Reference	Country Where the Study Was Performed	Sensitivity (Number of Samples tested)	Commentary
Saliva	RT-hnPCR(L gene)	[3]	France, Madagascar, Cambodia	70.2% (*n* = 84)	A sensitivity of 100% was observed for three serial daily saliva samples per patient with encephalitic rabies.Liquid saliva (a volume of 200 µL for analysis) is preferable to saliva swab.
	RT-PCR(N gene)	[16]	France	30% (*n* = 37)	A volume of 200 µL was used for analysis. A proteinase K lysis step was included.
	NASBA(N gene)	[4], [5]	Thailand	75.8% (*n* = 62)	A volume of 200 µL was used for analysis.
	RT-nPCR(N gene)	[17]	India	37.5% (*n* = 24)	Pool of three saliva samples collected at intervals of 4–5 h.Confirmation of rabies with reference technique (FAT) is not mentioned in this study.
	RT-qPCR (SYBR Green)(N gene)	[17]	India	75% (*n* = 24)	Pool of three saliva samples collected at intervals of 4–5 h.Confirmation of rabies with reference technique (FAT) is not mentioned in this study.
Skin biopsy	RT-hnPCR(L gene)	[3]	France, Madagascar, Cambodia	98.3% (*n* = 60)	Collected at the nape of the neck, containing hair follicles.A sensitivity of 100% (*n* = 33) was obtained at hospital admission and 97.2% (*n* = 36) at hospital discharge or death.
	RT-PCR(N gene)	[7]	Brazil	70% (*n* = 10)	Most neck-skin biospy samples were collected post-mortem (8/10).
Extracted hair	NASBA(N gene)	[4], [5]	Thailand	50% (*n* = 26)	At least 20 hairs have to be collected.
Urine	RT-hnPCR(L gene)	[3]	France, Madagascar, Cambodia	9.5% (*n* = 63)	The difference in sensitivity found in these studies seems to be due to difference of volume of tested samples.At least 1 mL has to be collected and analyzed (as amount of viral RNA found in this samples is low).
	NASBA(N gene)	[4], [5]	Thailand	39% (*n* = 41)	
CSF	RT-PCR(N gene)	[16]	France	8% (*n* = 12)	A volume of 200 µL was used for analysis. A proteinase K lysis step was included.
	NASBA(N gene)	[4], [5]	Thailand	43.3% (*n* = 30)	A volume of 200 µL was used for analysis.

FAT, fluorescent antibody test; hnPCR, hemi-nested PCR; L, polymerase; N, nucleoprotein; NASBA, nucleic acid sequence-based amplification; nPCR, nested-PCR; PCR, polymerase chain reaction; qPCR, real-time PCR; RT, reverse transcription.

**Table 2:** National Recommendations from the NRC Rabies, WHO-CC for Reference and Research on Rabies, France, Concerning Biological Samples Collected for Diagnosis of Human Rabies.

Intra-Vitam Diagnosis of Human Rabies					
Samples	Sensitivity Considering the Clinical Evolution of the Patient (in Days following the Onset of Symptoms)		Comments^a^	Storage	Technique (Reference)
	0–8 Days	>8 Days			
Saliva (1 mL or saliva swabs)	High	High	At least three saliva samples collected at intervals of 3–6 hours.Liquid saliva is preferable to saliva swabs.	−20°C/−80°C	RT-hnPCR [3]
Urine (at least 1 mL)	Low	Low	At least three urine samples collected in an interval of 3–6 hours.	−20°C/−80°C	RT-hnPCR [3]
Skin biopsy (diameter of 4 mm, total volume of 20 mm^3^)	High	High	Skin biopsy collected at the nape of the neck, with hair follicles, using biopsy punch (Stiefel).	−20°C/−80°C	RT-hnPCR [3]
Serum (500 µL)	Low	Average	Sample collection can be repeated, depending on the length of survival period (1–2 samples per week).	+4°C/−20°C	RFFIT [31] and/or ELISA [3], [6], [33]
CSF (>300 µL)	Low	Average	Sample collection can be repeated, depending on the length of survival period (1–2 samples per week).	−20°C/−80°C	RT-hnPCR [3]; RFFIT [31] and/or ELISA [3], [6], [33]

Post-Mortem Diagnosis of Human Rabies				
Samples	Sensitivity	Comments	Storage	Technique (Reference)
Brain biopsy	High	Brain biopsy collected via the orbital route with Tru-Cut biopsy needles for soft tissues with manual clip (Allegiance) or via the occipital route using lumbar puncture needles.	+4°C/−20°C	FAT [28]; RTCIT [29]; WELYSSA [26], [27]; RT-hnPCR [3]
Skin biopsy (diameter of 4 mm, total volume of 20 mm^3^)	High	Skin biopsy collected at the nape of the neck, with hair follicles, using biopsy punch (Stiefel).	−20°C/−80°C	RT-hnPCR [3]

a A minimum of three serially collected saliva samples and one skin biopsy from the nape of the neck are required for intra-vitam diagnosis of human rabies. This permits a sensitivity of 100% in patients presenting with encephalitic rabies (≥98% with skin biopsy alone and 100% with serial saliva samples). Skin biopsy samples can be collected irrespective of the time after the onset of symptoms. Analysis of additional samples may be advantageous. It includes RNA detection in serially collected urine and CSF samples, and rabies antibody measurement in serum and CSF. Rabies antibody detection is variable but increases with time after onset of symptoms (>8 days) and can be informative with atypical or non-classical rabies. After collection, all samples have to be stored at −20°C/−80°C before shipment. Storage at +4°C or on ice should not exceed a few hours (<6 h). All specimens should be shipped at −20°C or −80°C.

FAT, fluorescent antibody test; RFFIT, rapid fluorescent focus inhibition test; RTCIT, rapid tissue culture infection test; RT-hnPCR, reverse transcription hemi-nested polymerase chain reaction; WELYSSA, ELISA dedicated to lyssavirus antigen detection.

**Table 3:** National Recommendations from the WHO-CC for Research and Training on Viral Zoonoses, Thailand, Concerning Biological Samples Collected for Diagnosis of Human Rabies.

Intra-Vitam Diagnosis of Human Rabies					
Samples	Sensitivity Considering the Clinical Evolution of the Patient (in Days following the Onset of Symptoms)		Comments^a^	Storage	Technique (Reference)
	0–8 Days	>8 Days			
Saliva (1 mL or saliva swabs)	High	High	Collected once daily. Repeat testing if result is negative.Liquid saliva is preferable to saliva swabs.	−20°C/−80°C	NASBA [11], [18]; TaqMan RT-PCR [43]
Urine (at least 2 mL)	Average	Average	Collected once daily. Repeat testing if result is negative.Fresh urine is preferable to stored samples.	−20°C/−80°C	NASBA [11], [18]; TaqMan RT-PCR [43]
Extracted hair (at least 20 follicles)	Average	Average	Collected once daily. Repeat testing if result is negative.	−20°C/−80°C	NASBA [11], [18]; TaqMan RT-PCR [43]
CSF (1 mL)	Average	Average	Collected once daily. Repeat testing if result is negative.	−20°C/−80°C	NASBA [11], [18]; TaqMan RT-PCR [43]

Post-Mortem Diagnosis of Human Rabies				
Samples	Sensitivity	Comments	Storage	Technique (Reference)
Brain biopsy/autopsy	High	Brain biopsy collected via the orbital route with use of Tru-Cut biopsy needle (if full autopsy cannot be performed). In case of standard autopsy procedure, sample of brainstem region must be included.	+4°C/−20°C	FAT [28]; NASBA [11], [18]; TaqMan RT-PCR [43]

a Samples from all sources should be examined simultaneously due to the intermittent shedding of virus. If not possible, at least thrree of four less-invasive samples—saliva, extracted hair follicles, and urine—should be collected. Repeat testing if the first result is negative and test for other etiologic agent(s). RNA detection may be less sensitive on patient with paralytic rabies. Detection of rabies antibody is not sensitive in serum (approximately 25%) and CSF (none was found positive even 3 weeks after onset) in Asian dog related cases. Therefore, antibody detection-based assays are not recommended in this protocol. All specimens should be transported immediately (on ice) and if not possible, should be stored at +4°C for no longer than 24 h or at −20°C or −80°C (preferable) if storage time is longer.

FAT, fluorescent antibody test; NASBA, nucleic acid sequence-based amplification; RT-PCR, reverse transcription polymerase chain reaction.

## Preservation of Samples

It is also essential to mention the importance of the conditions required for the shipment and preservation of specimens, especially when molecular techniques will be used. The use of glycerol as a transport medium is convenient for brain biopsy samples [6]. However, the maintenance of a cold chain (+4°C or −20°C) remains essential when using samples for the intra-vitam diagnosis of rabies [6]. These preliminary steps still pose a challenge in most developing countries. A reliable system for transporting samples from hospitals to reference laboratories has to be organised. The use of commercial storage media for nucleic acid [8], RNA extraction reagents [9], or filter paper technology [10] might be good alternatives.

## Preparation of Samples

Different protocols have proved effective for the preparation of samples for viral RNA detection by nucleic acid extraction and reverse transcription (RT) [3], [11]–[14]. However, as emphasised in previous publications [2], [6], strict quality control measures must be followed to avoid false positive or negative results. This includes the use of appropriate controls for each step of the process (e.g., using endogeneous RNA as beta-actin or ribosomial 18S RNA, or other spiked and/or synthetic controls). This applies to RT, polymerase chain reaction (PCR), and other molecular techniques such as nucleic acid sequence-based amplification (NASBA) and loop-mediated isothermal amplification (LAMP) techniques. Positive results of viral RNA in samples should be confirmed by sequencing to avoid false positives, although it is recognised that sequencing capacity may not yet be available in most developing country settings. This also permits genotyping of viral strains, which is useful for epidemiological surveillance.

## Choice of the Most Appropriate Molecular Techniques for Viral RNA Detection

Molecular techniques for the detection of rabies and rabies-related lyssaviruses usually use PCR methods targeting the viral nucleoprotein gene as indicated by Fooks et al. [2]. However, another region of the lyssavirus viral genome, the polymerase gene, can also be used. This gene encompasses several nucleotide blocks (such as block III) that have been highly conserved during lyssavirus evolution [15]. A new RT, hemi-nested PCR (RT-hnPCR) protocol employing this region was developed and standardized in three laboratories, one in France and two in developing countries where rabies is still endemic (Cambodia and Madagascar) [3]. Using this RT-hnPCR, it was possible to diagnose human rabies (intra-vitam and post-mortem) using saliva, urine, and skin biopsy specimens collected from a large cohort of patients with suspected rabies (51 patients enrolled and 425 samples collected and analyzed). In this study, a sensitivity of more than 98% was obtained using single skin biopsy sample, irrespective of the time of collection (i.e., from 1 day after the onset of symptoms to just after death), and of 100% when at least three successive saliva samples per patient were analyzed. This study confirmed and complemented other results obtained by RT-PCR [7], [16], [17] and by NASBA techniques [11], [18], but not all were mentioned by Fooks et al. [2]. All these results indicated that it is now possible to perform a reliable intra-vitam diagnosis of human rabies by collecting serial saliva samples [3], [5], [11], [17], [18] or simply a skin biopsy from the nape of the neck, which shows a high sensitivity even when taken right at the time of admission [3], [7]. RNA detection in skin biopsy tissues is more sensitive than in extracted hair follicles (98% versus 53%), although the latter is easier to perform [4], [5]. These studies provide a clear demonstration of the feasibility of using molecular methods for human rabies diagnosis in developing countries (Brazil, Cambodia, India, Madagascar, Thailand, etc.). These techniques have also the potential to improve rabies surveillance, particularly in countries where the incidence is expected to be high, and the only evaluations have been made using mathematical models reliant on extrapolation [1], [19].

## Rabies-Specific ELISA Techniques

ELISA techniques can be used for the detection of rabies antigens or anti-rabies IgG. These tests are rapid, cheap, easy to use, and safe because they do not require the use of infectious virus, making them suitable for use in developing countries.

### Antigen Detection

Apart from the recently developed direct rapid immunohistochemical test (dRIT) [20] and rapid immunodiagnostic test (RIDT) [21] for rabies antigens detection, a validated ELISA for the rapid post-mortem detection of antigen in brain samples is also available but was not mentioned by Fooks et al. [2]. It has been evaluated under field conditions in different countries [22]–[25] and its use is recommended by the WHO [6]. Another reliable, rapid, and transferable ELISA method named WELYSSA is designed to detect lyssaviruses belonging to all seven genotypes circulating in Europe, Africa, Asia, and Oceania [26], [27]. Performance of both of them have been compared to techniques of reference for rabies diagnosis, i.e., the gold standard fluorescent antibody test (FAT) [28] for rabies antigens detection and the rapid tissue culture infection test (RTCIT) [29] for viral isolation (which should replace the mouse inoculation test [30], according to WHO recommendations). Results demonstrated a high concordance in terms of sensitivity and specificity [22], [26], [27].

### Antibody Detection

An ELISA for antibody detection is also recommended by the WHO for testing serum or CSF from patients with rabies encephalitis, but mainly for assessment of the immune status of vaccinated patients [6] especially when the reference methods, the rapid fluorescent focus inhibition test (RFFIT) [31] or fluorescent antibody virus neutralization test (FAVN) [32], cannot be performed, as is usually the case in developing countries. Although not mentioned in [2], several previously published studies have compared the sensitivity of such methods to standardized reference methods [33]–[35]. In particular, a second generation ELISA test, the Platelia Rabies II (Bio-Rad), has been favourably evaluated in a multicentric study [33] and it is used in several developing countries (Vietnam, Cambodia, Senegal, Morocco, Madagascar) [36]. This shows that these methods can be easily transferred and used in laboratories that do not have the facilities for tissue culture or viral production techniques.

## Conclusion

As underlined by Fooks et al. [2], the development of new molecular tools for the detection of rabies virus, such as microarrays for lyssavirus detection [37], [38] or antibody titrations using lentiviral pseudotypes [39] are aimed at improving rabies surveillance and control activities. However, their applicability in laboratories in most rabies-endemic countries is debatable. On the other hand, validated, robust techniques already exist and should be transferred to these regions, monitored by quality control and regular interlaboratory evaluations. These include methods for viral isolation using RTCIT [29], and for antigen detection with ELISA tools [26], [27], dRIT [20], RIDT [21], and the gold standard FAT technique [28]. International programmes of networking, collaboration, training, and proficiency testing of these techniques should be further encouraged [40], [41]. Molecular diagnostic tools such as RT-PCR, real-time PCR, or other techniques such as NASBA [11], [18] or LAMP [42] should be made available more widely for the intra-vitam and post-mortem diagnosis of human rabies in rabies-endemic countries. An example of successful sharing and transferring of these molecular techniques between laboratories in both developed and developing countries has already demonstrated its feasibility [3]. The choice of the most relevant biological samples to collect and analyze remains essential and has to be clearly defined. We therefore provide recommendations from two reference laboratories concerning biological samples collection for human rabies diagnosis (Tables 2 and 3). In particular, analysis of three serially collected saliva samples and one skin biopsy taken from the nape of the neck offer the highest level of sensitivity. Analysis of other samples may improve sensitivity (Tables 2 and 3). Stringent quality control measures are required to avoid false positive results due to the high sensitivity of PCR and NASBA techniques [6]. As underlined by previous publications [2], [13], another major limitation is the lack of international standards for sensitivity determination of these techniques, making comparisons difficult. Until these standards are developed, we strongly urge that only molecular techniques that have been evaluated in local laboratories in rabies-enzootic countries are used on appropriate samples for human rabies diagnosis [6]. Proposing a list of technological parameters characterizing these methods independently from the description of biological samples and laboratory environments for which they are designed could result in a misrepresentation of the future development of human rabies laboratory diagnosis in the developing world.
